# PS‐SAM: A Mixed Methods Study to Understand Current Practice and the Facilitators and Barriers to the Utilisation of Psycho‐Social Stimulation Interventions in Severe Acute Malnutrition

**DOI:** 10.1111/mcn.70135

**Published:** 2025-11-05

**Authors:** Mike Kalmus Eliasz, Dolce Advani, Melissa Gladstone

**Affiliations:** ^1^ Department of Women and Children's Health, Institute of Life Course and Medical Sciences University of Liverpool Liverpool Merseyside UK

**Keywords:** child health, early child development, malnutrition, psychosocial

## Abstract

Psycho‐social stimulation interventions, recommended in the WHO guidelines for severe acute malnutrition (SAM), have been demonstrated to improve neurodevelopment and growth. However, interventions which have proven effective in clinical trials are resource‐intensive and may be challenging in many contexts. This study aimed to explore facilitators, barriers and the existing provision of psycho‐social stimulation interventions. We undertook a survey targeting practitioners across the globe who are involved in SAM care and/or programme management. We then undertook 12 semi‐structured key informant interviews with practitioners from diverse professional contexts. We transcribed and coded interviews using a deductive approach based on the Consolidated Framework for Implementation Science Research (CFIR). We gained 42 responses from 18 countries for our survey with 29 respondents including psycho‐social stimulation interventions in their SAM programmes. Our 12 key informant interviews described several barriers (financial, physical, and human resource limitations, prioritisation of life‐saving care, and staff beliefs) as well as some potential facilitators (inclusion in guidelines, enjoyment for staff and parents, and emerging evidence of benefits in terms of short and long‐term outcomes). This multi‐country mixed methods study revealed that there are very heterogeneous patterns around the implementation of psycho‐social interventions in children with SAM. Our study has demonstrated the perceived challenges by professionals involved in SAM care of the feasibility of implementing interventions from research trials. Pragmatic studies are needed which also include an assessment of implementation to enhance an understanding of what might drive uptake. Limitations of our study include a potential respondent bias and small sample size.

## Summary

- Implementation of psychosocial stimulation interventions for children with severe acute malnutrition is heterogenous with different levels of intervention by site and context delivered by different staff groups with no universal understanding on what it entails.
- Gold standard psycho‐social stimulation interventions for children with SAM are not feasible in all settings due to a range of factors particularly human and financial resource limitations and relative prioritisation of immediately lifesaving care.
- Facilitators of psycho‐social stimulation interventions include it being prioritised by policy makers through inclusion in national guidelines and that staff and parents can derive enjoyment from it.
- There are still significant gaps in terms of understanding feasibility and how to implement these interventions in a range of settings.

## 1 Introduction

Severe acute malnutrition (SAM) is estimated to affect 13.7 million children worldwide and is associated with a markedly increased risk of mortality in under 5's (WHO, UNICEF, & WBG. 2023). The key focus of care over recent decades has been to increase coverage and simplify both diagnosis and treatment through community management of acute malnutrition (CMAM), simple screening through assessment of mid upper arm circumference (MUAC) and provision of ready to use therapeutic foods (RUTF) (Lenters et al. 2016).

Surviving episodes of malnutrition is associated to a broad spectrum of adverse outcomes across the life course, including impaired neurodevelopment and an increased risk of non‐communicable diseases (Kirolos et al. 2022; Lelijveld et al. 2016). Evidence suggests that psycho‐social stimulation interventions when provided alongside existing medical and nutritional care, can potentially improve neurodevelopment and growth. With a systematic review stating ‘the evidence supporting the recommendation of psychosocial stimulation for children with SAM is not only sparse, but also of very low quality across important outcomes’ (Daniel et al. 2017). At the time of writing only three studies exist showing potentially statistically significant results, all initiated in a structured manner in an inpatient setting—albeit with different levels of intensity or programmes provided.

Original studies in Jamaica conducted in the 1980s using the ReachUp model, an intensive programme, showed improved motor and language development during long term follow up (Grantham‐McGregor et al. 1982). One intervention in Bangladesh also using the intensive ReachUp model has showed improved mental development, gross motor functioning and growth (only weight for age z‐scores)(Nahar et al. 2009). A further randomised controlled trial in Ethiopia using trained playleaders working with families over 6 months showed improvement in gross and fine motor skills, but no improvement in linear growth or nutritional outcomes (Abessa et al. 2019). One further published study in Malawi using a more feasible and less intensive approach showed no evidence of benefit on neurodevelopmental and nutritional outcomes and a further study in Nepal was abandoned due to limited application of the treatment protocol and poor data collection (Daniel et al. 2021). There are ongoing studies to gather further evidence on this topic (Bossard et al. 2024; Jensen et al. 2024; T. T. Tessema et al. 2024; Tesfalem T. Tessema et al. 2019).

Despite the limited number of studies, WHO recommends incorporating psycho‐social stimulation into care for children with SAM. The 2023 updated guidelines define this as the ‘sensory information received from interactions with people and environmental variability that engages a young child's attention and provides information; examples include talking, smiling, pointing, enabling, and demonstrating, with or without objects. This also includes responsive feeding as a part of responsive caregiving’ (WHO 2023b). These recommendations are also included in the WHO's *Pocketbook on Hospital Care for Children* and other SAM guidelines and a UNICEF quick guide on early childhood development as part of emergency nutrition responses (UNICEF 2023; WHO 2013). Several national SAM guidelines also advocate for psycho‐social stimulation, ranging from comprehensive packages to simply ensuring facilities for play are available (Malawi 2016; MOH 2017). However, the guidelines are varied in their specificity and often cover a wide range of overlapping elements beyond the recent WHO definition including play therapy, physical therapy, mental health, psychological, family, and wider social support.

Some programmes, such as ReachUp initiative used in Jamaica and Bangladesh combine elements such as home visits and group sessions every 1–2 weeks over 6–12 months. These programmes provide specific toys for certain ages of children and an evidence based package of counselling and education for parents and caregivers of malnourished infants (Hossain et al. 2024). Information on coverage of these interventions is scarce with only one published study available focused on coverage in Malawi (Daniel. 2019).

Rising food prices, ongoing conflicts, climate change and the economic fallout of the COVID‐19 pandemic are leading to a stagnation of progress in addressing acute malnutrition, with a clear association between worse childhood nutrition outcomes and exposure to economic shocks (Silva et al. 2025). These challenges are also placing increased strain on both human and financial resources (WHO 2023a). Traditional SAM programme metrics focus on coverage, mortality rates, and anthropometric measurements. Targets are set to increase coverage and simplify treatment regimens including moving children with uncomplicated SAM to community‐based treatment through CMAM programmes (Lenters et al. 2016). Despite being in guidelines, these programme metrics do not include the availability psychosocial stimulation interventions. To implement the guidelines we need to understand both current practice and the perceived barriers and facilitators to psycho‐social interventions in SAM care in real world settings. This will enable future research studies to study what is feasible and may help to shape guideline development, policy, and programme management.

## 2 Methods

### 2.1 Study Design

We aimed to understand reported current practice and the perceived facilitators and barriers to psycho‐social stimulation interventions in the management of SAM across a range of different contexts. Our study consists of two components; firstly, a survey which was distributed on social media (twitter and LinkedIn) and through professional networks of humanitarian, nutrition and early childhood development communities between July 2021 and September 2022 (detailed below) and secondly, semi‐structured key informant interviews (KII) with health professionals, nutritionists, and programme managers. The use of qualitative methods was to enable a ground up approach rather than presume the views and perceptions of participants.

We chose, however, to use both a deductive and inductive approach to our qualitative study using the Consolidated Framework for Implementation Research (CFIR) to understand the facilitators and barriers to implementation of psycho‐social stimulation interventions in SAM treatment across different contexts. The CFIR framework is an implementation science framework that has been utilised in a range of contexts to understand influences on intervention implementation in health systems at multiple levels (Damschroder et al. 2009). The CFIR was chosen over other theories and frameworks for qualitative implementation research due to its pragmatic nature as well as its prior validation as a tool for understanding the implementation of different interventions. The CFIR was updated in 2022 after the study had commenced, therefore the original 2009 iteration was used for this study (Damschroder et al. 2022).

The 2009 iteration CFIR consists of 5 domains and 37 constructs, which in our case, have provided a framework to understand the barriers and facilitators to psycho‐social interventions in the management of SAM (Damschroder et al. 2009). The CFIR domains relate to Innovation Characteristics, Outer Setting, Inner Setting Characteristics of Individuals and Process (Table 1).

**Table 1:** CFIR constructs and definitions.

Domain	Definition and constructs
Innovation characteristics	The characteristics of the ‘thing’ being implemented. Constructs include: intervention source, evidence strength, relative advantage, adaptability, trialability, complexity, design quality and cost.
Outer setting	The wider economic, political and social context in which the implementing entity resides. Constructs include patient needs, cosmopolitanism, peer pressure, external policies and incentives.
Inner setting	Features specific to the implementing entity itself. Constructs include structural characteristics of the entity, networks and communication, culture, implementation climate (absorbative capacity of an organisation and shared receptivity of individuals to an intervention)
Characteristics of individuals	Individual behaviour and attitudes towards an intervention. Constructs include knowledge and beliefs, self‐efficacy, individual stage of change and identification with organisation and other personal attributes
Process	The actual process of implementing an intervention. Constructs include planning, engaging, executing and reflecting and evaluating.

Before study commencement, we undertook formative discussions with stakeholders to map key organisations involved in the care of children with SAM so that we could identify the best routes for data collection (Appendix 1). Key groups identified included intergovernmental organisations, research institutions, NGOs and government health facilities.

The final paper was checked against the COREQ checklist to ensure that it was presented in line with the standards for such research (Appendix 3).

### 2.2 Study Participants and Sampling Design

This study protocol was developed during the COVID‐19 pandemic which required data collection to be remote and collected online. Participants were restricted to healthcare professionals (nurses, therapists, doctors and nutritionists) directly treating patients with SAM and nutrition programme managers directly managing individual programmes. Including programme managers alongside clinicians was based on formative discussions so that data could also be captured from those working in CMAM programmes, who had less direct clinician involvement with individual patients but still had an important role in decision making of the programme. Participants must have engaged in direct patient care or SAM programming in the field in the past 3 years, to ensure that information provided was current. All responses were collected both from the survey and KII in English.

Purposive sampling was employed to select participants to the study. Stakeholders identified via stakeholder mapping (Appendix 1) were contacted directly or through appropriate professional networks such as the Emergency Nutrition Network (ENN) and Early Childhood Development Action Network (ECDAN) and invited to complete the survey with a request to share it with their contacts. Those who completed the survey were then directly approached via email and asked to participate in KII to provide further insights on their responses. The initial targeted sample size based on similar studies utilising CFIR was between 10 and 20 interviews with the aim of reaching thematic saturation, this was constrained by the research teams time and resource availability.

### 2.3 Data Collection

The survey utilised the Qualtrics platform and was hosted on the University of Liverpool servers (Qualtrics 2021). It gathered key demographic information about respondent and details about their workplace such as geographical location, patient volume and operational personnel. The survey featured both checkbox responses and free text sections to ascertain the current provision of psycho‐social stimulation interventions in each setting, with opportunities for respondents to provide additional contextual information qualitatively as free text.

A topic guide (Appendix 2) for KII was developed and piloted using two key informants before conducting the final set of interviews. The interviews were conducted online using Zoom by MKE, the lead of this study and a paediatrician with experience in nutrition policy and managing children with SAM, between June 2021 and July 2022 during the COVID pandemic, and audio recorded at the time.

### 2.4 Data Analysis

Survey data was anonymised and analysed utilising Microsoft Excel to extract key descriptive information about the respondents and what was offered in the projects.

Before data analysis the KII's were transcribed verbatim into word documents utilising the services of a professional transcription service. The approach to data analysis involved thematic analysis and deductively coding the data based on the CFIR framework, with any themes identified that did not fit the coding framework to be coded inductively. (Gale et al. 2013). The data was coded by MKE with second coding by DA utilising the NVIVO software package.

MKE and DA initially familiarised themselves with the data from the first three transcripts jointly coding them according to the CFIR domains and constructs and looking for any themes that needed to be coded inductively as they fell outside the CFIR. After this exercise we were able to adapt the CFIR to our needs, predominantly by removing codes for constructs that were not relevant and adding in facilitator and barrier codes, we proceeded to code the interviews our adapted coding framework. We then summarised the identified the relevant facilitators and barriers by CFIR domain and construct as well as disability specific findings which did not neatly map onto the wider CFIR codes.

### 2.5 Ethical Considerations

The study received ethical approval from the Institute of Life Course and Medical Science Research Ethics Committee at the University of Liverpool on 13th July 2021 (reference 8682). A consent form was embedded into to the survey and participants in KII were sent study information sheets and consent forms before the interviews. All transcripts were anonymised with only broad aggregate data available for analysis and not identifiable to the level of an individual respondent.

## 3 Results

### 3.1 Survey

The survey (Table 2) received 42 responses from 18 countries in Africa, Asia and South America. The type of role which respondents came from varied and included doctors (33.3%), nutritionists (23.8%) project managers (23.8%) with a range of other professional roles (some of the nutritionists and project managers held other professional qualifications e.g. nursing).

**Table 2:** Summary of survey response data (42 respondents total).

	N (%)
Role	
Doctor	14 (33.3%)
Nurse	2 (4.8%)
Nutritionist	10 (23.8%)
Project manager	10 (23.8%)
Other	6 (14.3%)
Type of project^a^	
Inpatient facility	26 (61.9%)
Community based project	23 (54.8%)
Outpatient hospital facility	20 (47.6%)
Other	3 (7.1%)
Project type^a^	
Government	28 (66.7%)
NGO	16 (38.1%)
UN entity	9 (21.4%)
Other	5 (11.9%)
University	4 (9.5%)
Project offers some kind of psychosocial stimulation^a^	N = 31 (73.8%)
Play interventions (e.g. toys and group play available)	28 (90.3%)
Physical/occupational therapy	16 (51.2%)
Mental Health Support for parents	14 (45.2%)
Formal psycho‐social stimulation package	14 (45.2%)
Who delivers the intervention^a^	N = 29
Parents	15 (51.7%)
Nurses	14 (48.3%)
Community health workers	12 (41.4%)
Doctors	10 (34.5%)
Psychologists	4 (13.8%)
How often is the intervention offered	N = 29
Multiple times per day	2 (6.9%)
Daily	16 (55.2%)
Weekly	8 (27.6%)
Monthly	2 (6.9%)
Parental education once only	1 (3.4%)
How long is the intervention offered for	N = 28
Once only	2 (7.1%)
Duration of inpatient stay	13 (46.4%)
2 months	3 (10.7%)
3 months	2 (7.1%)
4 months	1 (3.6%)
4–6 months	0
Between 6 months and 1 year	2 (3.6%)
Greater than 1 year	5 (17.9%)
Believe the intervention offered improved survival	N = 29
Definitely yes	19 (65.5%)
Probably yes	7 (24.1%)
Might or might not	2 (6.9%)
Probably not	1 (3.4%)
Definitely not	0
Believe the intervention offers improved child development	N = 29 =
Definitely yes	21 (72.4%)
Probably yes	6 (20.7%)
Might or might not	2 (6.9%)
Probably not	0
Definitely not	0

a Respondents were able to select multiple options for this question.

The majority of participants, 31 (73.8%) offered some form of psychosocial stimulation intervention with the most common offer being availability of play interventions (toys and group play space) 28 (90.3%) with some offering multiple interventions such as physical therapy or mental health support for parents but only a smaller proportion of those offering interventions offered psycho‐social stimulation interventions as a formal package (structured activities as part of routine patient care offered to all patients). Interventions varied in frequency and duration with only 13 (46.4%) of those offered longer durations (greater than once or inpatient stay only) these participants were more likely to be using a formal package such as ReachUp.

In the qualitative responses from the survey, most participants mentioned that interventions most frequently included just having play materials or a space available rather than a structured package of psycho‐social interventions. Some participants mentioned that they used multidisciplinary professional approaches including physiotherapy and occupational therapy, psychology and social workers delivering interventions. Most interventions were delivered by parents often with support of nurses or community health workers.

All respondents did state that psycho‐social stimulation interventions should be a priority. But on questioning a majority stated that human and financial resource constraints combined with it being a relatively low priority for policy makers offered barriers to expansion or initiation of such a package.

### 3.2 Outcomes From Key Informant Interviews

A total of 12 semi‐structured qualitative interviews were conducted with key informants who agreed to participate in the study (Table 3). Key informants included those working in the Democratic Republic of Congo, Ethiopia, Gambia, Kenya, Malawi, Pakistan, Rwanda, South Sudan, Sudan, Tanzania, West Africa (regional programme Mali & Niger) and Zimbabwe with summaries provided on these programmes and participants in Table 3. Three respondents were nurses, two were nutritionists/programme managers, one was a physiotherapist and six were doctor's paediatricians and generalists.

**Table 3:** Key informant interviews.

	Country	Role	Project type	Use of psychosocial intervention in the treatment of SAM (Yes/No)	If yes, is it offered Formally/Informally?	Brief description
Interview 1	South Sudan	Nurse	Humanitarian	No	—	Humanitarian inpatient and CMAM project in a rural location. During the interviewee's time at the project in the hospital, there was nothing for psychosocial stimulation activity as the whole unit was a tent. There were plans to build something for this soon and dedicate a play area for such activities. There was also an IYCF Counsellor in the team, but they mostly focused on feeding rather than messaging on this topic.
Interview 2	Democratic Republic of Congo	Nutritionist	Humanitarian	Yes	Formally	A large nutrition project including inpatient and outpatient care across multiple sites in a district. Nursing staff training on the importance of play and stimulation and provided education to mothers. Education was also provided to doctors on importance of such interventions. Stimulation and play activities on the ward three times a day. Play and stimulation interventions continued in the community following discharge.
Interview 3	Malawi	Nurse	Government Facility	Yes	Formally	A large inpatient nutrition ward. This intervention was introduced as part of a research study, it usually takes place in a less populated ward of the hospital – organised as a play area for children. Nurses interact and play with children with available toys such as dolls, toy cars. Mothers also take part in play activities and are encouraged to continue these activities at home. However, this is still not considered as a main priority in the treatment, rather the main focus is to save lives and there are limited resources to continue the intervention following the study.
Interview 4	Kenya	Paediatrician	Government	No	—	An Inpatient ward at a large tertiary urban hospital. No formal provision of psycho‐social intervention in the treatment programme, some limited ad hoc volunteer led play activities primarily from faith‐based organisations. Poor mental health cases are referred for additional support only when its considered ‘extreme’ to psychology. The interviewee was not familiar with the term ‘psycho‐social stimulation’.
Interview 5	Sahel	Physical Therapist	Regional Humanitarian	Yes	Formally	Regional pilot programme in inpatient settings across the Sahel implemented in multiple nutrition hospital facilities in West Africa in humanitarian contexts. The project was from a rehabilitation focused NGO. It utilised physical therapists to train health agents in physical therapy and rehabilitation techniques. Patients received inpatient rehabilitation plans and packages followed up by community health workers who were trained. This project was part of a pilot and was evaluated for developmental outcomes showing benefit.
Interview 6	Gambia	Nurse	Government/Research	No	—	There is no formal/informal psychosocial intervention in this organisation for treatment of SAM. The interviewee was not familiar with psycho‐social intervention in nutrition and explained that their main focus was direct management of malnutrition and its complications.
Interview 7	Pakistan	Paediatrician	Government	No	—	Inpatient government teaching hospital nutrition ward. There was only verbal advice on managing malnutrition and parenting but no psycho‐social stimulation offer with the focus on feeding practices.
Interview 8	Sudan	Doctor/Advisor	NGO/Humanitarian	Yes	Was formally offered for some time—6 months—12 months	Part of a larger multi‐country ECD package offered in different sites by a major NGO, the interviewee was directly involved in Sudan, but model is being piloted and trialled in Rwanda, Haiti, Guatemala and Ethiopia not only in children with SAM. There are impact evaluations, but not published in the formal literature, comparing outcomes with a control group who only received CMAM care only. The intervention in Sudan involved direct integration in CMAM with 10 group parenting sessions. It was the only intervention done exclusively in an outpatient context. The intervention involved parental education, toy making and child play. There were benefits shown compared to children receiving CMAM in terms of developmental outcomes and caregiver behaviours but was not perfect research design. The model is currently being adapted to be implemented by other faith leaders in Haiti.
Interview 9	Ethiopia	Medical Doctor	Research/Government	Yes	Yes, but mostly only offered to those who the clinician feels may most benefit from it.	A government hospital with the intervention offered in a research context. The interviewer stated as a resource limited country where play/psychosocial stimulation is considered a luxury rather than a priority in SAM treatment. Hence, the stimulation is unstructured, and perhaps informal with a room available. Those identified as likely to benefit from more intervention receive formal package of care including support from child and adolescent psychiatry and a graduate student providing a formalised stimulation and play intervention. It is usually not offered more than 3–5 percent of their patients. They are working on plans to expand this specialised offer of care in the region in collaboration with the Ministry of Health.
Interview 10	Zimbabwe	Paediatrician	Government	Yes	Informally	The intervention team consists of a counsellor who visits every day and a social worker who conducts play activities in a boardroom once a week together with parents and voluntary staff. The activities were based on the WHO guidelines, and take place in a dedicated cubicle which was sourced through crowdfunding and recently renovated, it consists of arts, crafts, TV and more.
Interview 11	Rwanda	Programme Manager	NGO	Yes	Informally	Community focused nutrition NGO working on malnutrition in the community through treatment and education of parents. This not a major part of their programme but sometimes they allocate an individual and some toys and allocating a staff member to support with a priority of making the child feel ‘involved’. They are now trying to add a greater ECD component to their programme through parental education as funding has become available.
Interview 12	Tanzania	Doctor	Research/Government	Yes	Formally	Inpatient government hospital facility. The intervention was introduced and developed in the context of a research study. During the inpatient stay a curriculum is followed a delivered by nurses. The first week's focus is on nutrition completely and from the second week onwards, they introduce psychosocial stimulation and provide knowledge about it to the caregivers and children. They are made aware of the importance of communication and play in this context with some resources.

The KII's provided a heterogenous range of views from different contexts as well as those who were working at different stages of implementation of psycho‐social stimulation interventions within the context of SAM programming. Some described having no overall intervention and some described having a comprehensive package of interventions. Furthermore, there existed a spectrum of understanding regarding the scope of psycho‐social stimulation interventions, spanning from maternal mental health, play therapy and physical therapy to parental education. This range also included various direct nutrition interventions, such as feeding practices.

Facilitators and barriers were identified for 4 of the 5 CFIR domains (Table 4). Given the study was conducted at different places along the implementation journey for different sites and interviewees it was challenging to infer process specific facilitators and barriers as the interviewees were not all implementing psycho‐social interventions at their workplaces. Findings for each domain as well as disability specific findings are elaborated below.

**Table 4:** Facilitators and barriers identified from key informant interviews.

CFIR domain	Facilitator	Barrier
Innovation characteristics	Implementing psycho‐social interventions does not necessarily have to cost more money and may prevent future problems. The evidence that does exist suggests a positive benefit and there is evidence from programmatic data. Can be implemented in different ways with some designs complementing work on malnutrition unit making for a more pleasant environment. If shown to prevent future complications may prove cost saving by preventing recurrence.	Perceived to require additional staff and resources which are not always available. Strength of evidence to support this is not perceived as that strong and challenging to develop good evidence in contexts where interventions needed the most. Ability to adapt this intervention to fit into the basics of care for children with acute malnutrition. There is a lack of consensus on exactly what is constituted by psycho‐social stimulation interventions in SAM care.
Outer setting	Incorporation into government guidelines and WHO guidelines guides practice. Engagement of ministries of health in ECD and incorporation into planning from national to regional level	Overall funding for healthcare system is limited in many settings. Interventions take place in contexts of low health literacy and awareness of ECD, with limited availability of resources including for transport to attend follow up. Relatively underprioritized at the national level by health policy makers
Inner setting	Potential to enhance working environment for staff and patients. Opportunities for staff development. Incentivising in local clinical practice. Seeing children engaging in psycho‐social interventions is rewarding in its own right for both staff and mothers. Staff and parental awareness of the issue and need for intervention.	Lack of space, funds and staffing. Caregivers having more immediate priorities such as preserving livelihoods and surviving in challenging humanitarian contexts. Staff prioritising immediately lifesaving care. Rationale for intervention poorly understood by families and staff, with limited access to education.
Characteristics of individuals	Individual staff buying into the intervention. Seeing the value of the intervention and advocating its importance.	Lack of individual staff's knowledge and belief on the intervention. Perceptions of the intervention not being priority relative to other things.

### 3.3 Innovation Characteristics

The understanding of what was specifically included in a psycho‐social stimulation intervention and its components varied among respondents (Table 4). Most participants acknowledged that they felt there was evidence supporting its implementation and that they felt there was positive impact on children's outcomes.

Several interviewees described how they believed that there was potential for psycho‐social stimulation interventions to positively affect the care environment for children, their families, and staff. The belief that there was potential to improve outcomes through these interventions was identified as a facilitator ‐ particularly as it provided immediate benefits, not just long‐term ones.

I think something that was important I found really interesting is that the nurses who are implementing this really enjoyed to implement this activity. They saw the significance and they felt like there was a huge contribution to the health care that was being provided. The only challenge is always the workload.

(KII 3 Nursing Supervisor, Malawi)

I was thinking is just like, the whole inpatient care can be quite traumatising for mothers and children, it can also be quite traumatising for staff, so if they see that mothers and children are having some fun, that might also help put a smile on their face because it is tough work and they see lots of children die.

(KII 1 Nurse, South Sudan)

Some KIIs involved participants who had previously been involved in research studies. The standard of interventions which have been provided in prior trial conditions (e.g. weekly intensive play sessions and follow up over up to 18 months) were seen by those who had not been involved as relatively resource intensive, with not all projects being able to offer the kind of long term follow up that was offered in trial contexts. The more formal interventions that KIIs mentioned that were offered across sites were heterogeneous, often utilising different members of the multidisciplinary team when available, but all were dependent on resource availability for example the intervention in Sudan used nurses, but the organisation had used community health workers in another context (Table 3).

Except for one KII, who mentioned a research study they were involved in, all other KIIs mentioned that the contexts where they were providing stimulation interventions were initiated in inpatient settings, rather than through a community‐based outpatient programme.

### 3.4 Outer Setting

Interviewees described the wider challenges of the contexts where they are working and where SAM is prevalent. Issues raised included the relatively low prioritisation of early childhood development by policy makers, by paediatricians and nurses in government health facilities.

I think it has to come from the top it has to come from the Ministry of Health to say that this is the essential part of recovery, and all the hospitals will have to have some form of play therapy introduced and maintained in their facilities.

(KII 10, Paediatrician, Zimbabwe)

The challenges that come with providing healthcare where poverty is prevalent was also mentioned by healthcare professionals when asked about the feasibility of regular follow up. With doctors and nurses perceiving that it was likely that there were significant opportunity costs for carers to attend follow up appointments.

If it is far and they cannot afford the money to come for their appointments, and they're not receiving these food ‐ sometimes is very difficult as that makes a lot of them miss their appointments.

(KII 6, Nurse, Gambia)

SAM does not occur in resource rich environments and healthcare services in these settings are often under‐resourced, necessitating prioritisation of resources for services.

if we aligned it with other healthcare system or with other routine system, then again, additional manpower has to be employed, so that they can have enough time to stimulate to provide stimulation and other routine care.

(KII 9, Doctor Ethiopia)

Interviewees highlighted the importance of guidelines to changing practice and the inclusion of psycho‐social stimulation in national malnutrition guidelines as a potential facilitator of implementation of psycho‐social stimulation interventions.

We have that is the paediatrics protocol, Kenya paediatrics protocol, there are some 10 steps that are there in the management of a severe acute malnutrition period. Patient. So if those were to be included as part of the management in the booklet, that would increase the number of people who are able to practice them that way.

(KII 4, Paediatrician, Kenya)

### 3.5 Inner Setting

When discussing the actual psycho‐social interventions which could be provided, a prominent theme that emerged from nurses and physicians concerned resource availability. Some respondents described how they needed to prioritise routine and ‘lifesaving’ aspects of malnutrition care which are often more acutely needed than those aspects that might relate to longer term outcomes. It was obvious from the KII's who provided direct patient care, that in resource constrained environments, immediate lifesaving care took precedence, often overshadowing Early Child Development (ECD) interventions which were perceived as optional. Respondents in humanitarian contexts with food insecurity and conflict, such as in South Sudan especially reflected on this.

I wouldn't say a priority, I wouldn't prioritise them over treating infections or treating dehydration of the other things but it's definitely it has to be an essential parts of our treatments.

(KII 10, Paediatrician, Zimbabwe)

I think that's definitely a priority but oftentimes the issue is they say we're understaffed and there are kids that need lifesaving, immediate lifesaving interventions.

(KII 3 Nurse, Malawi)

The KIIs from Malawi, Kenya, Zimbabwe and South Sudan highlighted the relative priorities of caregivers emphasising that caregivers often face challenging circumstances, particularly poverty and lack of transportation. Engaging in follow‐up care can entail sacrifices such as lost income or balancing other caring responsibilities alongside caring for a child affected by SAM. Prolonged follow up with frequent sessions may not be feasible for all families. Suggestions were made by KIIs to be able provide long term psycho‐social interventions they would need to move follow up closer to families in the community or to offer interventions on discharge from hospital or as part of routine outpatient care.

### 3.6 Characteristics of Individuals

In several contexts, some KIIs described how successful implementation often hinged on the dedication of key individuals or the availability of specific professionals. In three different KII's access to individual physical therapists, psychologists and occupational therapists enabled a more comprehensive package of care, with perceived better outcome. Although recognising resource constraints better availability of allied health professionals in these settings could serve as a facilitator.

Furthermore, effective leadership and awareness of the value of these interventions is essential. Currently, some KIIs described how this drive is not consistently fostered by senior project staff. It was clear from the KIIs, that in many settings, there remains a gap in staff awareness regarding the value of these interventions, with some perceiving that further training and explanation could serve as a potential facilitator particularly in existing nutrition units such as those in South Sudan, Zimbabwe and Kenya.

I've also trained lots of health workers in Malawi on caring for children with severe acute malnutrition and this bit doesn't really come out.

(KII 3 Nursing Supervisor Malawi)

Beliefs of caregivers came out as a key area for work given that play is not seen as medicine in a traditional sense. The need to see benefits for parents' factor significantly in their engagement with treatment.

I think because you also require commitment and if mum is busy working and has also other siblings to take care of to convince her that actually spending a set amount of time just doing play activities, it might be also quite difficult. So probably they also need to really buy into the idea that that's what's making their children better as well as giving them medication and giving them plumpy nut or anything else.

(KII 10, Paediatrician, Zimbabwe)

They need to be convinced a bit more as to why it's important. And because if the benefits are not immediate, chances of them continuing with it after are not very high.

(KII 4, Paediatrician, Kenya)

Most of them they will come and receive the treatment. When they see there is improvement, they will not come back again. Until when things get worse, then they decided to come ‐ that's the problem, until it gets better, then, they will just miss their appointments.

(KII 7, Nurse, Gambia)

### 3.7 Process

Our survey and KIIs have primarily centred on current practice rather than the intricacies of implementation processes. However, insights from medical and nursing respondents in Malawi, Zimbabwe and South Sudan indicated that staff across the multidisciplinary team generally found satisfaction in participating in the implementation of these interventions where they were available. Local factors influencing the implementation process and the rationale behind them varied when described by KIIs, making generalisations difficult. Through the KIIs, it emerged that the more comprehensive intervention packages were typically funded directly by specific organisations or implemented for research purposes for example the interventions in Sudan, Ethiopia, the Sahel and Tanzania.

### 3.8 Disability Specific Findings

It is acknowledged that children with disabilities face a heightened risk of malnutrition and may benefit from interventions to maximise their developmental capabilities (Engl et al. 2022). During the key informant interviews, participants discussed the extent this was factored into interventions and whether any additional support was provided. Whilst some respondents could cite individual cases such as cerebral palsy, or groups of patients they had encountered, none of them reported offering a standalone interventions or specific additional services beyond the existing standard of care in their respective programme.

Some of these babies with disabilities they already have trouble with breast feeding and considering this gap of 0–6‐month infants unless they get severely malnourished that's when they come to the stabilisation centre, we don't do any screening it is easy to miss those that are born with disabilities.

(KII 1, Nurse, South Sudan)

KIIs recognised that a greater provision for patients with co‐morbid disabilities given their specific vulnerabilities could be helpful. The did acknowledge however, that resource constraints limited the ability to offer more for these patients. All respondents did express a desire to be able to do more for this specific group of inpatients.

this group of children, they come, obviously, they come and they stay for longer periods of times, as compared to children without neurological disability about a third of the patients that we admits will have neurological disability and majority, it will be cerebral palsy and they come with terrible problems with feeding their nasogastric tube fed for a prolonged period of time, it takes us time to get them back to the swallowing that they had when they were at home as well…. so, we're not too sure what's we know that they probably need special treatment, but we're not too sure what that special treatment should be. So that's also something that we are trying to partner up with different charities to try to develop the chapter in the guidelines, which particularly deals with combined malnutrition and disability as well.

(KII 10, Paediatrician, Zimbabwe)

## 4 Discussion

This study, to the best of our knowledge, is the first multi‐country survey to explore both current practices and facilitators and barriers to the implementation of psycho‐social stimulation and related activities in SAM programming. From our wide reaching, albeit limited in size, survey, it was clear that there is significant variation in terms of what is offered. We also were able to do in depth interviews with professionals from a range of countries and professionals. These in‐depth interviews allowed us to identify facilitators and barriers to such interventions and identify further areas of work.

Psycho‐social interventions are recognised as important by those looking after children with SAM, but they exist as part of a hierarchy of need and are by necessity ranked lower than immediate lifesaving. Psycho‐social interventions like many other in low and middle income countries, are often limited by the availability of resources both human and financial (Kruk et al. 2022). The current nutrition funding crisis as official development assistance is cut and supply chains for treatments such as RUTF come under increasing strain the challenge of basic care provision is likely to get worse (Osendarp et al. 2025). Under investment in tackling acute malnutrition will make scaling up the use of psycho‐social stimulation interventions incredibly challenging but there may be ways to do so without overburdening systems under increasing strain.

Firstly it is necessary to recognise that all programmes can do something, the most common intervention in both the survey and KII's was availability of toys and play spaces. Whilst this did not correspond to the intensity of intervention offered in formal clinical trials, with several respondents stating this would not be feasible in their context, it is still a step in the right direction (Grantham‐McGregor et al. 1982; Nahar et al. 2009). Improving care environments for children, caregivers and staff for which play is part of doing so should be done independently of whether an intervention is shown to have benefits for survival or growth. Interviewees repeatedly highlighted the traumatic nature of working in nutrition care and that this can make that work less distressing. There is already good evidence from a number of settings of the benefits of play as part of care of sick children without it necessarily being a structured activity (LeVieux‐Anglin and Sawyer 1993; Yogman et al. 2018).

Secondly for any intervention to succeed it needs to be able to overcome financial barriers and opportunity costs to caregivers especially if it lengthens inpatient stay or requires additional outpatient follow up. This came through from multiple interviewees and aligns with evidence from localised feasibility studies in Ethiopia and Tanzania (Jensen et al. 2024; T. T. Tessema et al. 2024). To overcome this staff and caregiver understanding and seeing the value and benefits of these interventions is key, as opposed to formal research evidence and this was reflected also in the evidence from the local feasibility studies. There may be multiple approaches to achieving this from staff mentoring and support, to incorporating into health promotion messaging. Additionally, there are likely lessons to be learned from peer support methods commonly used for mental health support in nutrition activities, bringing groups of caretakers and mothers together around this activity may not necessarily require additional staff time if caretaker led combing peer support and interacting with children (Haithar et al. 2018; Undlien, Viervoll, & Rostad. 2016).

In the current constrained funding environment, there is a tension between funding for research and implementation, this will require more efficient use of resources to generate evidence. Two interviewees possessed evaluations on interventions which are not publicly available, organisations should be encouraged to share this and help grow the evidence base on what works and allow learning across organisations. Trials that do take place should include a strong implementation science component not just to understand the efficacy of the intervention but also how it can be implemented in different contexts such as that which is being done with the BRIGHT‐SAM study in Tanzania, the MSF led STIMNUT study, the Tamba‐SAM study in Zimbabwe and the CO‐SAM randomised clinical trial in Kenya, Zambia and Zimbabwe (Bossard et al. 2024; Bwakura‐Dangarembizi et al. 2025; Jensen et al. 2024; Kabongo et al. 2025). All of these studies are also incorporating caretaker voices as part of their study design which should enable interventions to be developed that respond to their priorities.

A few respondents stated that greater prioritisation by policy makers both in terms of integrating into local guidelines and resourcing was essential to driving change. This aligns with other studies on scaling up similar mental health and psycho‐social interventions which highlighted the importance of buy in from policy makers (Jordans and Kohrt 2020). This leads to potential areas for further research including a review of national SAM guidelines to see both whether it is included at the national level and the degree of specificity in the guidelines. In additional interviewing nutrition policy leads on their perspectives of the relative prioritisation of psycho‐social interventions.

Finally, most of the uncomplicated SAM care worldwide now takes place in the community or outpatient context through CMAM programming (Mdege et al. 2023). Whilst one KII respondent did offer an integrated ECD and SAM package through a CMAM programme the remainder were all either offered exclusively in or initiated in inpatient settings, meaning that a relatively small proportion of children being treated for SAM are likely benefitting from these interventions. This means that future studies should place an emphasis on incorporation within routine CMAM programming for which there is little evidence. Additionally many countries struggle with high rates of default from nutrition programming for a wide variety of factors many of which were identified as barriers in our interviews (Mwima et al. 2025). Whilst currently very little research on this topic it would be valuable to explore how these interventions can be synergistic with others to tackle default rates, as children from default receive no benefit from either the malnutrition treatment or psychosocial intervention.

### 4.1 Limitations

The study had several limitations methodologically that may limit its generalisability to all contexts. There was potentially a degree of bias in response to both the survey and in the sampling of those who took part in the key informant interviews —given that those interested in psycho‐social interventions were potentially more likely to respond. We were unable to undertake any kind of systematic sampling to estimate the coverage of psycho‐social interventions within SAM programming globally. Clearly, more survey responses might have allowed for disaggregation of results by professional cadre to see if there were differences in perspective. Our survey was only able to be conducted in English and therefore likely not to capture responses from a range of non‐English speaking countries with a high burden of SAM.

The research team recognise that a limitation of this study is that as it was designed and mostly conducted during the COVID‐19 pandemic. This meant it was not feasible to capture the voices of caretakers in this study. Their voices would also be critical to understanding the facilitators and barriers to implementing psychosocial stimulation interventions for children with SAM.

Finally, the lead author who conducted the interviews for this study has worked directly caring for children with SAM in both humanitarian and non‐humanitarian contexts. This has potentially introduced a degree of reflexivity bias into the analysis, even with the utilisation of a second coder to interpret the interviews, his experiences will shape the interpretation of this data.

## 5 Conclusion

Evidence and guidance exists to support the use of psychosocial stimulation interventions as part of the care of children suffering with acute malnutrition (UNICEF 2023). Despite this not all children are able to benefit from them, with implementation as demonstrated by our study varying significantly between settings, and many of the interventions that are being implemented in routine care are different from those in trial conditions that generated the evidence to recommend their use. Whilst these interventions exist as part of a hierarchy of need with immediate life saving care taking priority a key finding of our study is that they can enhance the care environment and make it less traumatic not only for patients but also for staff too. We Programme managers, ministries of health, donors and technical agencies should encourage it because it is the right thing to do as part of providing family and child centred care and improving staff wellbeing regardless of the impact on growth or nutritional outcomes.

Researchers and funders going forward should continue to prioritise a strong implementation focus in all future studies on this topic. There needs to be a focus on how interventions can be resource multipliers, such as through improving staff morale and delivery without increasing the demands on precious human or financial resources. Finally, there is a need to understand how these interventions can be brought out into the community what the facilitators and barriers are to integrating into large CMAM programmes so that all children can benefit from these interventions.

## Author Contributions

M.K.E. designed the study with support and guidance from MG and conducted the interviews. Interviews and survey data were coded and analysed by M.K.E. and D.A. The final manuscript was prepared by M.K.E. All authors reviewed, edited and approved the final manuscript.

## Conflicts of Interest

The authors declare no conflicts of interest.

## Supporting information


**Appendix 1 –** Stakeholder Mapping. **Appendix 2 –** Topic Guide. **Appendix 3** – COREQ checklist.

## Data Availability

The data that support the findings of this study are available on request from the corresponding author. The data are not publicly available due to privacy or ethical restrictions.
