# Supplementary material for: PS‐SAM: A Mixed Methods Study to Understand Current Practice and the Facilitators and Barriers to the Utilisation of Psycho‐Social Stimulation Interventions in Severe Acute Malnutrition
Source: Matern Child Nutr. 2025 Nov 5;22(1):e70135. doi: 10.1111/mcn.70135 (PMC12900080; doi:10.1111/mcn.70135)
Supplement: Supplementary file 1 — Appendix 1 – Stakeholder Mapping. Appendix 2 – Topic Guide. Appendix 3 – COREQ checklist. [file MCN-22-e70135-s001.docx]

**Appendix 1 – Stakeholder Mapping**

| **Implementers and service providers** | **Government stakeholders** | **UN Agencies** | **Research Institutions** | **Professional Networks** | **Donors** |
| --- | --- | --- | --- | --- | --- |
| iNGOs:   - **MSF** - **ACF** - **Humanity & Inclusion** - **Alima** - **Save the children** - **Medair**   Local NGOs  Individual implementers:   - Healthcare workers - Nutritionists - Programme managers - Community health workers | Ministries of Health  Ministries of Education | **UNICEF**  **WHO** | **Universities in Global North e.g. LSHTM etc..**  **Universities in Global South e.g. Addis Ababa University** | Nutrition:   - **ENN** - **SUN** - Global Nutrition Cluster   Early child development   - **ECDAN** | **BMGF**  **USAID**  ECHO  GIZ  **FCDO**  **World Bank**  Eleanor Crook Foundation |

*** Targets initially approached to disseminate survey to networks**

**Appendix 2 – Topic Guide**

**Topic Guide for interviews**

**Understanding use of psychosocial stimulation in severe acute malnutrition programming.**

**Research Team:**  Dr Mike Kalmus Eliasz (Academic Clinical Fellow, Global Child Health) and Professor Melissa Gladstone (Consultant in Paediatric Neuro-disability at Alder Hey Children’s Hospital and Professor in Neurodevelopmental Paediatrics and International Child Health at the University of Liverpool).

**Introduction**

**Introduce self and study**

- Thank you for agreeing to talk to me today
- My Mike Kalmus Eliasz, I work as an academic clinical fellow at the University of Liverpool and paediatric trainee. The aim of this study is to understand the current practice with regards to the use of psycho-social stimulation in the management of SAM and how to optimise it for future
- It is included in the current WHO guidelines for managing SAM.
- You may not have directly described these interventions as psycho-social stimulation they may be understood as potentially play interventions, parent and child group activities or integrated into mental health and psychological support interventions.
- The primary aim of this study is to better understand current clinical practice, and how it could be better integrated in the future to improve survival and wellbeing in SAM programming.
- There are currently a number of pilot programmes and research studies ongoing it is important to inform such projects with how programmes could work at scale from reviewing existing projects and talking to those with experience working in or managing SAM programmes such as yourself.
- Additionally there is increasing recognition of the importance of disability in both determining who develops SAM and their response to treatment. Given what is known about the potential for early intervention to improve functional outcomes these children may especially benefit from psychosocial stimulation interventions in SAM care. I hope to conclude the interview asking a few questions on this.

**Aims of the interview – what I’d like to discuss**

- Today I’d like to find out about your experience of working in or managing a SAM unit/project. What your experiences are of psychosocial stimulation or other similar interventions are in the context of managing SAM. Your beliefs on the importance of such interventions, their feasibility and potential barriers to their uptake. I will also ask a little bit about your experience of managing disability in these contexts.
- If you have not heard specifically heard of psychosocial stimulation or its role in management of SAM it is ok to answer I don’t know to questions.
- I would like to stress that there is no right or wrong answer to the questions I ask and all answers are valid.
- Some questions may seem repetitive so I apologise this is to allow to me understand your answers in depth.

**Context of the interview**

- Length – this interview will last a maximum of one hour but I am aiming to take about thirty minutes is that ok?
- Participation is completely voluntary in nature we can stop at any time and you can withdraw without needing to give me a reason.
- I will be asking about experiences and if you find any topics distressing or challenging to answer feel free to ask me to pause, move on to the next question or stop at any time.
- Are you happy for me to record this interview? I am recording to enable accurate transcribing of the interview and allow me to focus on conversation.
- Anonymity and confidentiality
  - All responses will be kept confidential and only accessed by myself and the immediate study team.
  - Results published will be anonymised with all identifying features removed so nobody will be able to identify you individually.
- Do you have any questions for me at this stage?
- Are you happy for me to start recording?

**Background, professional and project details**

- First of all can I ask a few questions about you, your professional role and the project you work/worked on?
- What is/was your job title?
- Where do/did you work?
- How long have/did you work there?
- What did your role involve in management of SAM?
- Can you tell me a little bit about the nature of the project? Was it inpatient or outpatient? The context in which it took place? How many patients were you seeing roughly?
- Can you describe how the project functioned, who was covered and how it was managed?
- Who delivered and worked on the project?

**I would like to ask a bit about psychosocial stimulation in the context of your project.**

- What do you understand by the term psychosocial stimulation is and its use in the context of SAM?
- If not familiar how about the role of play or mental health interventions?
- Can you describe the current offer in your current/former project?
- In a bit more detail
  - Who delivers? By parents, staff members
  - How is it delivered?
  - Resources required?
  - Time involved
- Do you think what you offer is sufficient or not and why?
- Would you like to offer more if it was an option?
- Outpatient?
- Do you think play and psychosocial interventions are a priority?

**Gold standard interventions**

- The evidence supporting psychosocial stimulation is based on two trials. One involved play and stimulation interventions for an hour every week over 2 years and the other 18 sessions over 6 months from admission to SAM programme to discharge. The interventions themselves were delivered by mothers but with close support.
- All programmes were started in hospital. They did show survival and cognitive benefits.
- Hearing this what comes to mind when thinking about potential interventions in your context?
- Do you think such a package would be feasible?
- What do you think would be the barriers to such an intervention? Feasibility, cost, interest, uptake.

**Optimal Interventions**

- In an ideal world what would you think is a feasible offer to improve child development in SAM projects?
- How do you think psycho-social care could be improved for children and their caregivers in these programmes?

**Disability**

- Finally I want to ask a few questions on disability in SAM programmes. This is not the primary objective of this study but is a secondary topic which we believe is connected to psychosocial care in SAM.
- What has been your experience of patients with disabilities in SAM programmes?
  - Did you specifically aim to identify them?
  - What if anything did you offer them different to other patients?
  - Do you think their needs were met?
- There is evidence from a number of setting that children with disabilities are at increased risk of SAM and evidence from high income settings shows that early multidisciplinary interventions can improve functional outcomes. What do you think should be being offered in an ideal world? How could the psycho-social needs of children and their caregivers with disabilities and SAM be better met?
- Do you see an appetite within your organisation for further work on this?

**Conclusion**

- Is there anything else you would like to add at this stage
- Do you have any questions for me?
- Thank you for taking the time to discuss
- End recording
- Reassure re confidentiality and anonymity
- Offer to send the results and follow up by email.

Appendix 3 – COREQ checklist

| **No** | **Item** | **Guide questions/description** | **Response** |
| --- | --- | --- | --- |
| **Domain 1: Research team and reflexivity** | | |  |
| **Personal Characteristics** | | |  |
| 1. | Interviewer/facilitator | Which author/s conducted the interview or focus group? | Mike Kalmus Eliasz |
| 2. | Credentials | What were the researcher's credentials? *E.g. PhD, MD* | Dr. |
| 3. | Occupation | What was their occupation at the time of the study? | Doctor/Research Fellow |
| 4. | Gender | Was the researcher male or female? | Male |
| 5. | Experience and training | What experience or training did the researcher have? | Author’s qualifications (mentioned above) are a representation of their relevant experience for this study. |
| **Relationship with participants** | | |  |
| 6. | Relationship established | Was a relationship established prior to study commencement? | Some interviewees were known to the interviewer in a professional capacity. |
| 7. | Participant knowledge of the interviewer | What did the participants know about the researcher? e*.g. personal goals, reasons for doing the research* | Some of the interviewees knew about participant in a professional capacity. Before commencing each interview, a brief introduction of the interviewer followed by a briefing about study objectives and the procedure of the interview was provided. This was also provided in the participant information leaflet while recruiting. |
| 8. | Interviewer characteristics | What characteristics were reported about the interviewer/facilitator? e.g. *Bias, assumptions, reasons and interests in the research topic* | The study goals were informed to the participants. The interviewer’s background and interest in early childhood development and interventions caused a lack of objectivity. This was reported as a researcher reflexivity and bias under the study limitations. |
| **Domain 2: study design** | | |  |
| **Theoretical framework** | | |  |
| 9. | Methodological orientation and Theory | What methodological orientation was stated to underpin the study? *e.g. grounded theory, discourse analysis, ethnography, phenomenology, content analysis* | *Thematic analysis*  (Independant qualitative analysis approach; no particular orientation followed) |
| **Participant selection** | | |  |
| 10. | Sampling | How were participants selected? *e.g. purposive, convenience, consecutive, snowball* | *Purposive* |
| 11. | Method of approach | How were participants approached? e*.g. face-to-face, telephone, mail, email* | *Approached via e-mail for online interviews* |
| 12. | Sample size | How many participants were in the study? | 12 |
| 13. | Non-participation | How many people refused to participate or dropped out? Reasons? | 0 |
| **Setting** | | |  |
| 14. | Setting of data collection | Where was the data collected? e*.g. home, clinic, workplace* | Interviews were conducted online where both the interviewer and interviewee were at different settings, for example, home or workplace. |
| 15. | Presence of non-participants | Was anyone else present besides the participants and researchers? | No |
| 16. | Description of sample | What are the important characteristics of the sample? *e.g. demographic data, date* | Country, profession/role, type of psychosocial/other project, organisation, duration. For maintaining confidentiality, no names have been reported |
| **Data collection** | | |  |
| 17. | Interview guide | Were questions, prompts, guides provided by the authors? Was it pilot tested? | The interview guide was created by the author (MKE) with additional review of the semi-structured guide from two academic clinical fellows. Prompts were used wherever needed in the interview(s). |
| 18. | Repeat interviews | Were repeat interviews carried out? If yes, how many? | No |
| 19. | Audio/visual recording | Did the research use audio or visual recording to collect the data? | Yes, audio recording used to transcribe the interviews at a later stage. |
| 20. | Field notes | Were field notes made during and/or after the interview or focus group? | Yes, mostly just for the interviewer’s reference. |
| 21. | Duration | What was the duration of the interviews or focus group? | On average : 30 to 45 minutes |
| 22. | Data saturation | Was data saturation discussed? | Briefly in the methods section. |
| 23. | Transcripts returned | Were transcripts returned to participants for comment and/or correction? | No |
| **Domain 3: analysis and findings** | | | |
| Data analysis | | | |
| 24. | Number of data coders | How many data coders coded the data? | Two |
| 25. | Description of the coding tree | Did authors provide a description of the coding tree? | All codes consisted of themes and sub-themes coded into the CFIR framework. |
| 26. | Derivation of themes | Were themes identified in advance or derived from the data? | Themes were derived in advance from the CFIR framework |
| 27. | Software | What software, if applicable, was used to manage the data? | NVivo Version 14.23.0 |
| 28. | Participant checking | Did participants provide feedback on the findings? | No |
| **Reporting** | | | |
| 29. | Quotations presented | Were participant quotations presented to illustrate the themes / findings? Was each quotation identified? e*.g. participant number* | Yes |
| 30. | Data and findings consistent | Was there consistency between the data presented and the findings? | Yes |
| 31. | Clarity of major themes | Were major themes clearly presented in the findings? | Yes |
| 32. | Clarity of minor themes | Is there a description of diverse cases or discussion of minor themes? | There was only one minor ‘divergent case’ out of all the interviews where the psychosocial package was performed in an outpatient setting. This has been mentioned in the main body of the paper. |
